# Change in pulmonary function tests and their relation to the serum levels of ceruloplasmin, copper and superoxide dismutase in patients suffering from Type-1 and Type-2 diabetes

**DOI:** 10.12669/pjms.39.3.6485

**Published:** 2023

**Authors:** Nida Lathiya, Qamer Aziz, Asher Fawwad, Iftikhar Ahmed Siddiqui, Abdul Basit

**Affiliations:** 1 Nida Lathiya, M.Sc., M.Phil., Ph.D. Assistant Professor, Department of Physiology, Baqai Medical University, Baqai Medical University, Karachi, Pakistan; 2 Qamer Aziz, M.B.B.S., M.Phil., Ph.D. Professor and Chairman, Department of Physiology, Baqai Medical University, Karachi, Pakistan; 3 Asher Fawwad, M.B.B.S., D.D.M., M.Phil., Ph.D. Professor and Head, Department of Biochemistry, Baqai Medical University, Research Director (Honorary), Department of Research, Baqai Institute of Diabetology and Endocrinology, Baqai Medical University, Karachi, Pakistan; 4 Iftikhar Ahmed Siddiqui, M.B.B.S., M.Phil., Ph.D. Professor and Chairman, Department of Biochemistry, Baqai Medical University, Karachi, Pakistan; 5 Abdul Basit, Professor of Medicine, Department of Medicine, Baqai Institute of Diabetology and Endocrinology, Baqai Medical University, Karachi, Pakistan

**Keywords:** Ceruloplasmin, Copper, Diabetes Mellitus, Pulmonary Function Tests, Superoxide Dismutase

## Abstract

**Objective:**

To correlate the serum levels of ceruloplasmin (Cp), copper (Cu), and superoxide dismutase (SOD) with pulmonary function tests (PFTs) in non-diabetics (controls) and patients suffering from Type-1 and Type- 2 diabetes.

**Methods:**

The comparative cross-sectional study of 348 participants was performed at the Baqai Institute of Diabetes and Endocrinology (BIDE) - Karachi, Pakistan, from February 2019 to September 2020. Individuals having diabetes-related complications, asthma, chronic obstructive pulmonary disease, chest infection, pregnant women and smokers were excluded. A total of 348 participants were included into three groups after signing informed consent. The control group had 107 non-diabetic participants, with an age range of 6 to 60 years. The diagnosed T1D group (n=107) had an age range of 6 to 25 years. While diagnosed T2D group (n=134) had an age range of 26 to 60 years. During the fasting state, anthropometric parameters, blood pressure, spirometry, and a venous blood sample (5ml) were collected to measure serum Cp, serum Cu, serum SOD, and HbA1c levels by using commercially available kits. The SPSS, version 21, was used for data analysis.

**Results:**

The reduced FVC (*p*-value <0.001), FEV1 (*p*-value <0.001), and PEFR (*p*-value <0.001) were found in both groups of diabetes. However, the lower levels of serum Cu (*p*-value <0.001), SOD (*p*-value <0.001), and significantly increased values of FEV1/ FVC (*p*-value <0.001) and Cp levels (*p*-value 0.030) were found only in T2D group as compared to T1D and controls. The study found no significant correlation of PFTs and serum Cp, Cu, and SOD levels in patients suffering from T1D and T2D.

**Conclusion:**

Hyperglycemia leads to more non-enzymatic glycosylation of tissue proteins that reflects reduced PFTs and increased Cp; particularly in T2D, which may alter lung tissue’s physiology. Moreover, the study showed no correlation of PFTs with the Cp, Cu, and SOD in patients suffering from T1D and T2D.

## INTRODUCTION

Hyperglycemia is one of the main markers for diabetes mellitus (DM) that damages glucose metabolic pathways and enhances auto-oxidative glycosylation and formation of free radicals. This produces toxic effects and disturbs the proper functioning of organs.^1^ The free radicals stimulate oxidative stress which decreases the lungs’ elastic recoil, pulmonary volumes, and performance of respiratory muscles.^2^ Spirometry is a non-invasive method that helps to evaluate pulmonary function tests (PFTs) that give information for any lung obstructive or restrictive disease.^3^

Copper (Cu) works in the catalytic action of superoxide dismutase (SOD) for cell protection; however, its instability disturbs the metabolic pathways and causes oxidative damage that leads to diabetes-related complications.^4^

Ceruloplasmin (Cp) is a copper-dependent ferroxidase enzyme that oxidizes ferrous iron (Fe2+) within the cells and delivers ferric iron (Fe3+) outside the cells. Therefore, higher levels of Cp and Cu were found based on the inflammatory machinery triggered by the autoimmune condition.^5^

The scope of this research is relatively new for the Pakistani population with the objective of estimating Cp, Cu, SOD, and PFTs in T1D and T2D. The data gathered can be used to improve the methods of treatment addressing people with T1D and T2D.

## METHODS

The participants; both genders, were taken from Baqai Institute of Diabetology and Endocrinology (BIDE) Karachi, Pakistan from February 2019 to September 2020. The ethical approval of this comparative cross-sectional study was obtained from the Ethics committee of Baqai Medical University (BMU) reference: BMU-EC/2018-03. Individuals having diabetes-related complications, asthma, chronic obstructive pulmonary disease, chest infection, pregnant women, and smokers were excluded. A total of 348 participants were included into three groups. The control group had 107 non-diabetic participants, with an age range of 6 to 60 years. The diagnosed T1D group (n=107) had an age range of 6 to 25 years. While, diagnosed T2D group (n=134) had an age range of 26 to 60 years. Informed written consent was taken from each participant after mentioning the significance of the study. The results were disseminated through hardcopy.

To calculate body mass index (BMI) (kg/m²), the participant’s weight (kg) was measured through a standard weighing scale while, height (cm) was measured in a standing upright position with their back to the ruler. The participants were instructed to remove their shoes and extra clothing. In a fasting state and sitting position, the blood pressure (BP) (mmHg) was measured by standard auscultatory method^6^ while, the pulmonary function tests (PFTs) were performed by portable spirometer- Model no: SP10BT, China. The sterilized disposable plastic mouthpiece was used for each participant to measure forced vital capacity (FVC) (%), forced expiratory volume in 1st second (FEV1) (%), FEV1 / FVC (%), and PEFR (%) three times at every two minutes intervals and took the best of three readings.^7^ The fasting venous blood samples were taken for estimating glycated hemoglobin (HbA1c (%) by Bio-Rad analyzer,^6^ serum Cp levels (mg/dl) by Immunoturbidimetric method^8^ on SelectraProS-Merck using Cp kit, DIALAB, Austria, the levels of serum Cu (μg/dl) by Photometric method^9^ on SelectraProS-Merck which automatically read the absorbance at 580 nm using Cu kit, DIA LAB Austria, and serum SOD levels (U/ml) using DR-200Bs Microplate Reader of Diatek (USA) by Enzyme-Linked Immunosorbent Assay (ELISA) via SOD kit, Bioassay Technology Laboratory, Korea.^10^

## RESULTS

**Fig.1:**
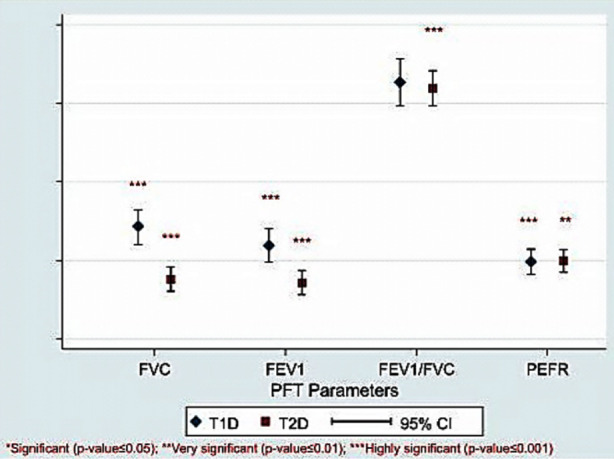
The comparison of PFTs parameters- FVC, FEV1, FEV1/FVC and PEFR in patients of T1D with T2D.

The results were analyzed on Statistical Package for the Social Sciences (SPSS) 21. Out of 107 healthy participants, 29 age-matched participants were compared with 107 diagnosed T1D and 78 age-matched participants were compared with 134 diagnosed T2D. A statistical value of *p*<0.05 was considered significant. On comparing PFTs parameters, the study found significantly lower values of FVC (*p*-value <0.001), FEV1 (*p*-value <0.001), and PEFR (*p*-value <0.001) in T2D as compared to T2D (Fig.1).

The Mann-Whitney test was used for comparing the mean differences of demographic and clinical factors in cases (T1D and T2D) and controls. The study observed highly significant values of Height (*p*-value 0.002), Weight (*p*-value <0.001), BMI (*p*-value 0.030), SBP (*p*-value 0.014), and HbA1c (*p*-value <0.001). While, significant lower values of FVC (*p*-value <0.001), FEV1 (*p*-value <0.001) and PEFR (*p*-value <0.001) in T1D as compared to controls. While in the T2D group, the study observed significantly higher values of Weight (*p*-value 0.018), BMI (*p*-value 0.020), HbA1c (*p*-value <0.001) and Cp (*p*-value 0.030). Whereas, significantly lower values of FVC (*p*-value <0.001), FEV1 (*p*-value <0.001), FEV1/ FVC (*p*-value <0.001), PEFR (*p*-value <0.001), Cu (*p*-value <0.001) and SOD (*p*-value <0.001) as compared to controls were observed (Table-1). The Spearman correlation was used between PFTs with other variables for patients of T1D and T2D.

**Table-I:** Comparison of demographic and clinical factors in T1D and T2D (n=348).

Characteristics	T1D	Controls	p-value	T2D	Controls	p-value
	
	Mean ± SD	Mean ± SD		Mean ± SD	Mean ± SD	
Age (years)	19.59 ± 4.93	18.89 ± 4.02	0.358	47.72 ± 9.51	46.13 ± 9.99	0.186
Height (cm)	166.16 ± 8.98	158.53 ± 11.71	0.002**	161.94 ± 9.22	159.47 ± 19.37	0.737
Weight (kg)	64.38 ± 12.74	53.70 ± 13.29	<0.001***	75.60 ± 12.65	71.94 ± 13.23	0.018*
BMI (kg/m2)	23.25 ± 3.87	21.24 ± 3.70	0.030*	28.89 ± 4.70	27.61 ± 5.26	0.020*
SBP (mmHg)	109.66 ± 12.88	103.05 ± 11.40	0.014*	116.11 ± 16.25	113.72 ± 17.10	0.256
DBP (mmHg)	72.41 ± 9.22	70.25 ± 7.50	0.316	78.05 ±11.10	75.26 ± 9.00	0.079
HbA1c (%)	9.81 ± 2.58	5.06 ± 0.65	<0.001***	8.94 ± 2.52	5.60 ± 0.70	<0.001***
Cp (mg/dl)	33.59 ± 5.37	32.78 ± 6.41	0.183	37.64 ± 6.80	35.60 ± 6.08	0.030*
Cu (μg/dl)	114.80 ± 31.65	112.48 ± 38.93	0.291	105.12 ± 18.20	127.96 ± 40.75	<0.001***
SOD (U/ml)	98.76 ± 189.81	177.24 ± 434.66	0.124	131.70 ± 119.05	183.07 ± 368.94	<0.001***
FVC (%)	48.75 ± 23.12	68.38 ± 19.28	<0.001***	35.29 ± 18.05	74.15 ± 16.80	<0.001***
FEV1 (%)	43.85 ± 22.54	68.00 ± 17.89	<0.001***	34.38 ± 18.32	61.04 ± 21.32	<0.001***
FEV1/FVC (%)	85.38 ± 31.89	92.40 ± 19.12	0.345	83.85 ± 26.17	76.64 ± 23.29	<0.001***
PEFR (%)	39.69 ± 16.95	66.67 ± 24.63	<0.001***	39.90 ± 16.87	51.12 ± 20.92	0.001**

p-value calculated using Mann Whitney test,

* Significant (p-value≤0.05),

** Very significant (p-value≤0.01);

*** Highly significant (p-value≤0.001)

In the T1D group, the study showed a significant positive correlation of FVC, FEV1, and PEFR with Age (r=0.337 p-value <0.001; r=0.233 p-value 0.016; r=0.337 p-value 0.001), Height (r=0.282 p-value 0.003; r=0.249 p-value 0.010; r=0.376 p-value <0.001), Weight (r=0.428 p-value <0.001; r=0.318 p-value 0.001; r=0.409 p-value <0.001) and BMI (r=0.395 p-value <0.001; r=0.272 p-value 0.005; r=0.287 p-value 0.003). Moreover, the FEV1 and PEFR showed a significant positive correlation with SBP (r=0.218 p-value 0.027; r=0.224 p-value 0.023) respectively (Table-II).

**Table-II:** Association of PFTs with other study variables in T1D

Variables	T1D							

	FVC		FEV_1_		FEV_1_/FVC		PEFR	

	r	p-value	r	p-value	r	p-value	r	p-value
Age (years)	0.337	<0.001***	0.233	0.016*	-0.068	0.486	0.331	0.001***
Height (cm)	0.282	0.003**	0.249	0.010*	0.008	0.933	0.376	<0.001***
Weight (kg)	0.428	<0.001***	0.318	0.001**	-0.082	0.404	0.409	<0.001***
BMI (kg/m2)	0.395	<0.001***	0.272	0.005**	-0.094	0.335	0.287	0.003**
SBP (mmHg)	0.162	0.102	0.218	0.027*	0.162	0.101	0.224	0.023*
DBP (mmHg)	0.002	0.984	0.025	0.802	0.097	0.331	0.124	0.213
HbA1c (%)	-0.025	0.800	0.029	0.769	-0.015	0.876	-0.070	0.473
Cp (mg/dl)	-0.011	0.915	-0.007	0.941	-0.004	0.965	-0.129	0.190
Cu (μg/dl)	0.024	0.810	-0.047	0.635	-0.095	0.337	-0.069	0.484
SOD (U/ml)	-0.106	0.280	-0.092	0.356	-0.165	0.092	-0.138	0.159

* Significant (p-value≤0.05);

** Very significant (p-value≤0.01);

*** Highly significant (p-value≤0.001), p-value calculated using Spearman correlation analysis.

In the T2D group, the study showed a significant positive correlation of FVC with Age (r=0.337 p-value <0.001), Height (r=0.282 p-value 0.003), Weight (r=0.428 p-value <0.001) and BMI (r=0.395 p-value <0.001) respectively. The FEV1 showed a significant positive correlation with Height (r=0.281 p-value 0.001) and BMI (r=0.217 p-value 0.012). However, the PEFR showed a significant positive correlation with Height (r=0.387 p-value <0.001) only. Whereas, significant negative correlation with Age (r= -0.247 p-value 0.004) and BMI (r= -0.202 p-value 0.019) was found (Table-III). No significant correlation was observed between PFTs with the levels of serum Cp, Cu, and SOD in patients with T1D and T2D (Table-II and Table-III).

**Table-III:** Association of PFTs with other study variables in T2D.

Variables	T2D							

	FVC		FEV_1_		FEV_1_/FVC		PEFR	

	R	p-value	r	p-value	r	p-value	r	p-value
Age (years)	0.337	<0.001***	-0.161	0.064	-0.006	0.949	-0.247	0.004**
Height (cm)	0.282	0.003**	0.281	0.001***	-0.021	0.818	0.387	<0.001***
Weight (kg)	0.428	<0.001***	-0.004	0.966	0.116	0.181	0.062	0.476
BMI (kg/m2)	0.395	<0.001**	-0.217	0.012*	0.107	0.218	-0.202	0.019*
SBP (mmHg)	0.162	0.102	0.069	0.429	0.100	0.252	-0.096	0.269
DBP (mmHg)	0.002	0.984	0.077	0.376	0.156	0.072	-0.004	0.962
HbA1c (%)	-0.025	0.800	-0.079	0.366	0.004	0.967	0.115	0.187
Cp (mg/dl)	-0.107	0.222	-0.112	0.204	-0.081	0.353	-0.080	0.361
Cu (μg/dl)	0.024	0.810	-0.012	0.893	0.004	0.967	-0.125	0.150
SOD (U/ml)	-0.106	0.280	0.006	0.941	0.103	0.235	-0.024	0.784

* Significant (p-value≤0.05);

** Very significant (p-value≤0.01);

*** Highly significant (p-value.0.001), p-value calculated using Spearman correlation analysis.

## DISCUSSION

During diabetes, various physiological changes occur in the lungs’ connective tissues.^2^ The present study showed significantly reduced values for FVC, FEV1, and PEFR for patients suffering from diabetes in comparison to the controls (Table-I) and significantly high reduction was observed in T2D as compared to T1D (Fig.1). Similar results were observed particularly with T2D group in other local studies done at Aga Khan University Hospital, Karachi,^11^ Baqai Medical University Teaching Hospital, Fatima Hospital, and Combined Military Hospital, Malir Cantt, Karachi^12^ and Mayo Hospital, Lahore.^13^ The results are similar for the T1D group from the study of Martin-Frias et al.^14^, however, none of the local studies reported these results. The more reduced values of FVC and FEV1 in T2D indicate the reduced alveolar epithelium thickening.^15^ While, the reduced PEFR showed the reduced expiratory muscle force-generating capacity of the lungs^16^ (Table-I). This indicates that in T2D there is more non-enzymatic glycosylation that stimulates increased oxidative stress in T2D.

The study shows higher mean values of HbA1c in patients having T1D and T2D than in controls (Table-I). This is supported by the few studies^11^,^17^,^18^ that reported increased enzymatic glycosylation of tissue proteins due to hyperglycemia; reflected by the reduced PFTs values. Anthropometric measures- Height, Weight, and BMI, were found significantly increased in both T1D and T2D groups as compared to the controls (Table-I). Multiple studies have showed similar results; local studies for T2D,^11^,^17^ and T1D study by Gajbhiye and Tambe supported this evidence.^18^

The study found decreased serum SOD in diabetic patients as compared with the controls (Table-I). The reference range i.e., 129-216 U/ mL, indicates the development of microvascular damage, which is supported by the study of Fei et al.^19^ However, this is not supported by the local study done in Rawalpindi, Karachi by Huma et al.^20^ On correlating the PFT parameters with SOD in patients suffering from T1D and T2D, no significant result was shown (Tables II and III).

Cu is required for the activity of SOD and cytochrome oxidase with normal mean levels from 70-140 μg/dL.^21^ In the current study, the mean Cu levels fall within the normal range. However, these levels were also found to be lower in diabetes which suggests that impairment of glucose tolerance can be secondary to copper deficiency.^22^ and may be due to the excessive loss of Cu in the urine^23^ compared to the controls (Table-I). On correlating the PFT parameters with Cu for both types of diabetes, no significant result was observed (Tables II and III).

The current study showed increased levels of Cp but within the normal range, i.e., from 20-40 mg / dL.^24^ (Table-I) The result is supported by the studies of William^22^ and Lee et al.,^25^ who showed that lungs are protected from oxidative stress by one of the antioxidant systems; however, elevated levels show the progression of microvascular diseases.^25^

The present study is relatively new for having normative data for Cp and SOD in Pakistani patients suffering from diabetes, especially T1D. Cp and SOD both work proficiently as an anti-oxidant which will be useful to apply as a treatment procedure to reduce the oxidative stress produced in patients suffering from T1D and T2D.

### Limitations:

The budget was constrained.

## CONCLUSION

PFTs were reduced in both groups of T1D and T2D as compared to the controls. Reduced FVC, FEV1, and FEV1/FVC ratio indicate obstructive ventilation. While, reduced PEFR showed reduced alveolar epithelium thickening and reduced expiratory muscle force-generating capacity of the lungs. This study shows that hyperglycemia leads to excessive non-enzymatic glycosylation of tissue proteins that reflects reduced PFTs and increased Cp, particularly in T2D. It will be useful to apply as an early treatment/management procedure to reduce the oxidative stress produced in diabetic patients. However, the Cp levels may not be correlated/associated with the PFTs changes in both patients suffering from T1D and T2D.

### Future Recommendations:

- The study can be further carried out from a vast population and other ethnicities to have more data.
- The role of Cu pathology in diabetes needs to be more certain with the evaluation of other metals.
- Clinicians especially diabetologists should introduce spirometry in OPDs as well as measure the serum Cp levels for the early detection of microvascular diseases, particularly in T1D patients.
